# Pancreatic senescence and its clinical manifestations

**DOI:** 10.1002/agm2.12095

**Published:** 2020-01-10

**Authors:** Lu Wang, Songbai Zheng

**Affiliations:** ^1^ Huadong Hospital Affiliated to Fudan University Shanghai China

**Keywords:** endocrine function, exocrine function senescence, pancreatic senescence

## Abstract

The pancreas is a vital organ which has both endocrine and exocrine functions and plays an essential role in food digestion and glucose metabolism. Pancreatic structure and function undergo a series of changes with aging and senescence. Pancreatic exocrine and endocrine function gradually change, which may lead to conditions such as dyspepsia and diabetes mellitus. Hence, clinicians need to be familiar with the characteristics of pancreatic senescence. This article reviews the manifestations of pancreatic senescence and its significance for clinical practice.

## 1 INTRODUCTION

The pancreas is situated deep in the retroperitoneum and has a complex histology, characterized by the unusual situation of endocrine and exocrine cells coexisting in the same organ. Acinar cells make up about 85% of the pancreas and are arranged in acini, small rounded clusters of secretory cells which empty into ducts, and which possess zymogen granules, which are functional units of digestive enzyme synthesis, storage, and secretion. Using the techniques of immunohistochemistry, acinar cells can be histologically labeled for lipase, amylase, trypsin, chymotrypsin, and elastase production. Each acinar compartment connects to the pancreatic duct system. The centroacinar cells are an extension of the most peripheral of the duct cells and partially cover the apical surface of each acini. Downstream of the centroacinar cells are the intercalated ducts; these converge and form the intralobular ducts and then the interlobular ducts, which in turn eventually drain into the main pancreatic duct. A much smaller percentage (only 2%) of the overall mass of the pancreatic gland is comprised of endocrine cells, organized into islets. These are comprised of at least four different types of endocrine cells: glucagon‐producing α‐cells, insulin‐producing β‐cells, somatostatin‐producing δ‐cells, and pancreatic polypeptide‐producing PP‐cells. Of these, β‐cells make up 60%‐80% of the islet cell population; it is dysfunction of these β‐cells that may cause diabetes mellitus (DM). Islets are fully developed several days after birth, and in the adult β‐cells cannot proliferate and differentiate. Both the morbidity and mortality of pancreatic diseases are increasing worldwide and are closely associated with changes taking place in the pancreas with aging. In this review, we examine these age‐related changes in the pancreas and how these affect clinical practice.

## 2 MORPHOLOGICAL CHANGES

### 2.1 General morphological changes

The pancreases of 112 autopsied adults without pancreatic diseases were weighed. Precise analysis of the data shows a significant descending trend in the quality of the pancreas from the fourth decade on.^1^ With the routine use of three‐dimensional computer tomography imaging to calculate the volume of the pancreas, it has been shown that in humans, pancreatic volume changes to a great degree with aging. Pancreatic volume reaches a maximal mean value of 78.85 cm^3^ in the third decade of life, with a mean volume of 73.50 cm^3^ for females and 84.21 cm^3^ for males (*P* = .006). Thereafter, pancreatic volume shows a definite downward trend with aging, which at the age of 70‐80 years shrinks to a mere 57.35 cm^3^ (*P* = .003).^2^ Janssen et al. have studied the texture of the pancreas by semi‐quantitative ultrasound endoscopic elastography. They found that in the young and middle‐aged group (i.e. age ≤ 60) and the elderly group (age > 60), the average strain values of the pancreases were 110.2 and 80.0, respectively (the values being inversely proportional to the hardness of the pancreas), indicating that the pancreas hardens in the elderly.^3^ Sato et al. used MRI to measure the anteroposterior diameters of the pancreatic head, body and tail of 115 subjects and showed that the pancreatic head, body and tail diameters of the four groups (aged 20‐39, 40‐59, 60‐79 and ≥80 years) were reduced successively (*P* < .05). The pancreatic tail showed the most significant reduction. The mean anteroposterior diameters of the four groups were 18.56, 16.83, 15.26 and 12.04 mm, respectively.^4^ Results from endoscopic retrograde cholangiopancreatography (ERCP) showed that, in the two groups of people aged <40 and ≥40, the mean diameter of the main pancreatic duct of the pancreatic head was 2.97 and 3.78 mm, respectively, while the mean diameter of the main pancreatic duct in the body of the pancreas in those two groups was 2.36 and 2.86 mm, respectively, indicating significant broadening of the main pancreatic duct in both the pancreatic head and body in the elderly. However, in the elderly, the length of the main pancreatic duct was found to be similar to that of younger persons.^5^ Hastier et al.^6^ also used ERCP to measure the dimensions of the pancreatic duct and found that the diameter of the pancreatic duct increased with age. Glaser et al.^7^ analyzed the results of pancreatic duct diameters, as measured by ultrasound, of 101 subjects aged 18‐91 years who had no pancreatic disease. The mean diameter of the pancreatic duct of all these 101 subjects was 1.9 mm and, further broken down into age groupings, was 1.5, 1.9 and 2.3 mm for the 18‐29, 40‐49 and ≥80 year‐old groups, respectively. As the maximum diameter of the pancreatic duct was less than 3 mm in all cases, the author surmised that if the pancreatic duct diameter is more than 3 mm, under no circumstances should pancreatic diseases be excluded in the elderly.

To sum up, the general morphological changes taking place during aging of the pancreas include: loss of mass, reduction of volume, hardening of texture, and dilatation of the pancreatic duct.

### 2.2 Microstructural changes

Riccillo et al. divided 18 Sprague‐Dawley (SD) male rats into three groups (4, 24 and 30 months old), and extracted pancreatic tissue for quantitative and qualitative analysis by HE and immunohistochemical staining.^8^ Significant fat infiltration in the exocrine part of the pancreas was observed in the aged rats, as well as the gradual replacement of acinar cells by adipose tissue. The interstitial fibrosis of the pancreatic tissue was remarkably aggravated, and the pancreatic ducts displayed adenomatous or cystic hyperplasia. The pancreatic duct was prominently dilated, the wall of which had thickened with aging. Detlefsen et al. used human specimens to study pancreatic aging, and found that the probability of pancreatic fibrosis in groups <60 years old and ≥60 years old was 10.3% and 62.0%, respectively, and that focal fibrosis mainly occurred in the peripheral pancreatic lobes, involving acinar tissue, small ducts and islets, and was frequently surrounded by lymphocytes. In addition, fibrosis‐related ductal papillary hyperplasia could be found in individuals over 60 years old.^9^ As a recent study has demonstrated, the attributes of an aging pancreas include: disordered arrangement of acinar epithelial basement membrane, increased fibrous tissue in the islets, disorganized structure and scattered arrangement of islets, papillary hyperplasia of duct epithelium, and gradual accumulation of lipid droplets in the matrix of the duct epithelial cells.^10^ The accumulation of lipid droplets in the matrix of the duct epithelial cells is also called duct epithelial cell fatty degeneration, which occurs mainly in the root tip area of large pancreatic duct epithelial cells. The number of fibroblasts and stellate cells is also increased in the pancreatic matrix. In addition, the authors found that focal fibrosis has a tendency to appear in the peripheral part of the pancreas, involving one or two more pancreatic lobes, and the acinar cells are gradually replaced by connective tissue. In addition, fibrosis is more evident around the small and medium‐sized ducts, the main manifestations of which are the gradual increase in basement membrane and connective tissue around the duct. The author has also suggested that papillary epithelial hyperplasia of the glandular ducts in the pancreatic lobes with obvious fibrosis may be a precursor of duct adenocarcinoma.^10^

Riccillo et al. studied the islet area, islet number and endocrine cell quantity in SD rats aged 4, 24 and 30 months. Their results showed that the islet area in the pancreatic endocrine tissue increased in the 24 and 30 months group compared with the 4 months group, but especially in the 30 months group (*P* ≤ .05).^8^ Compared with the 4 months group, the number of islets decreased in the 24 months group, but rebounded in the 30 months group, to a figure higher than that of both the 4 months group and the 24 months group, and indeed significantly higher than the 24 months group (*P* ≤ .05). There were no significant changes in A cells, D cells and PP cells among the three groups, and the number of cells (including volume density and cell density) showed similar changes as the number of islets among the three groups.^8^ A recent study found that in C57BL/6J mice pancreases, the expression of Ki‐67 and PDX‐1 decreased with increasing age, showing the deterioration of pancreatic proliferation and regeneration ability. However, the area and size of the islets enlarged with increased age. Compared with 2.5‐month‐old mice, the islet sizes of 21‐month‐old mice doubled, while the islet number remained unchanged; hence, it appears the older mice do have the ability to produce enough insulin to maintain normal blood glucose.^11^ Saisho et al.^12^ have studied the relationship between islet beta cells and aging in non‐diabetic human patients, and found that, with senescence, the size or area of beta cells significantly increased (*P* ≤ .01), and that the diameter of the beta cell nucleus significantly increased (*P* ≤ .0001), but that the number of beta cells remained unchanged (*P* = .9).

While the results of animal and human studies are not completely in agreement, the general conclusion is that while the number of islets and beta cells remains unchanged or increases with age, the area of islets and beta cells definitely increases. This may be a degenerative‐compensatory phenomenon in the aging process.

### 2.3 Ultrastructure changes

In rats, with the increase in age in months, the pancreas undergoes great changes. The volume of pancreatic acinar cells decreases and cellular vacuolization and nucleus pyknosis become more obvious. Mitochondrial dehydration, swelling and vacuolization become more evident. The expansion and scattered arrangement of the rough endoplasmic reticulum become evident, along with the growing number of lipid droplets and lysosomes. In addition, there is an obvious decreasing trend in the number of apical enzymogen particles. The intracellular rough endoplasmic reticulum of the pancreatic β cells expands and decreases in number. Swelling and vacuolation of mitochondria are obvious. Lipid droplets and lysosomes increase in number. The number of endocrine particles in the cytoplasm decreases, accompanied by larger halo inside these particles, which Chen et al. believe may lead to decreased insulin secretion.^13^ However, the ultrastructure of islet α cells do not change significantly with age.^14^ In another study, autophagy was found in islet cells of rats at 24 months old, since autophagosomes were observed. We believe that the appearance of autophagosomes is correlated with the degeneration of pancreatic endocrine function to some extent.^15^

## 3 ENDOCRINE FUNCTION

A study showed that the blood glucose peak and two hours postprandial blood glucose of rats increased gradually with the increase in age, accompanied by an obvious delayed insulin secretion peak. In vitro experiments using cells separated from rat islets found that both GSIR (glucose‐stimulated insulin release) and PSIR (palmitic acid‐stimulated insulin release) reduced with senescence.^16^ A Chinese study has suggested that both the basic secretion function of beta cells in rat islets and the secretion function after glucose stimulation decreases with age, accompanied by decrease of the insulin secretion peak and a gradually delayed peaking time, manifesting as obvious impaired glucose tolerance.^17^ Muller et al. performed an oral glucose tolerance test (OGTT) in 771 subjects aged from 20 to 96 years; those who took drugs or suffered from an illness which might affect glucose tolerance had been excluded. They found that blood insulin levels significantly decreased with increasing age, and the average insulin levels are 323, 267, 253 and 228 pM at ages 20‐39, 40‐59, 60‐79 and 80‐96 years, respectively, after the correction of relevant factors (*P* < .01).^18^

Both the basic secretory function and the glucose‐simulated secretory function of the islets decrease with age, which may be related to the following factors, in addition to the aforementioned beta cell structure degradation of the islet;^13^ (a) the expression of pdx‐1 gene, insulin gene, prohormone convertase 1/3 gene and other genes related to islet cell function are down‐regulated with aging, leading to islet cell function deterioration;^19^ (b) the number of glucose transporter 2 (GLUT2) decreases with aging, which reduces the transportation of glucose by the islet cell membrane, thus leading to impaired insulin secretion function stimulated by glucose (GSIS);^20^ (c) decreased uptake of calcium ions by cell endoplasmic reticulum in senescent islets leads to decreased insulin release;^21^ and (d) islet amyloid polypeptide (IAPP) accumulates in islet beta cells of the pancreas after aging, thus hindering the further release of insulin.^22^

## 4 EXOCRINE FUNCTION

Pancreatic exocrine function (both basic and reserve) deteriorates with age. A large population‐based study undertook the quantitative analysis of pancreatic exocrine function by measuring the fecal elastase‐1 (FE‐1) content of 914 healthy people aged 50‐75 years, the results of which showed that 11.5% of the subjects had pancreatic exocrine insufficiency (EPI), that is, FE‐1 < 200 μg/g, and that 5.1% had serious pancreatic exocrine insufficiency (SEPI), with FE‐1 < 100 μg/g. The incidence of EPI was 6.0%, 8.7%, 12.6%, 15.5%, and 13.4% (*P* = .005) in the groups aged 50‐54, 55‐59, 60‐64, 65‐69, and 70‐75, respectively. The incidence of SEPI was 3.0%, 4.7%, 5.0%, 7.2%, and 5.6%, respectively (*P* = .12). This indicates that the incidence of EPI and SEPI increases significantly with age.^23^ Another study measured the level of FE‐1 in 1105 patients without pancreatic disease but with symptoms of dyspepsia; the results showed that the level of FE‐1 was less than 200 μg/g in 10% of the elderly aged >70 years old, and <100 μg/g in 5% of those aged >70 years.^24^ Other reports have shown the incidence of EPI in the elderly to be as high as 21.6%.^25^

Torigoe et al.^26^ used dynamic magnetic resonance imaging of pancreatic biliary flow to evaluate the relationship between pancreatic exocrine function and aging. A total of 53 subjects were divided into three groups based on age (<40, 40‐70 and >70 years old). Pancreatic exocrine distance was divided into five grades according to the distance the pancreatic secretions flowed in the marked area by dynamic imaging during sweeping, in a sequence of 20 images: grade 0, no secretion of pancreatic secretions; grade 1, <5 mm; grade 2, 5‐10 mm; grade 3, 11‐15 mm; grade 4, >15 mm. The average secretion times of the three groups were 16.2, 11.9 and 4.8 (*P* < .001), respectively, indicating that the pancreatic exocrine function deteriorates significantly with senescence.

Ishibashi et al.^27^ divided 65 outpatient subjects free of bile pancreatic disease into three groups with different age ranges: group A (<40 years); group B (40‐65 years), and group C (>65 years); and collected pancreatic secretions every 10 minutes, one hour after the intravenous injection of secretin to promote pancreatic secretion. The total pancreatic secretion, bicarbonate secretion and pancreatic enzyme secretion of group C were significantly lower than those of group A and group B. Laugier et al. collected the duodenal fluid of 180 subjects aged 16‐83 with no pancreatic disease after intravenous injection of secretin to evaluate pancreatic exocrine function, the results of which showed that duodenal fluid production after the stimulation rose markedly with increase in age, up to 43 years, and then began to decline.^28^ Bicarbonate production began to increase, along with increase in age, up to 34 years of age, and then began to decline. The concentration and production of lipase, phospholipase and chymotrypsin showed a trend of gradual decline along with the increase in age after the age of 30 (*P* < .02).

Bulow et al. assessed secretin‐stimulated pancreatic secretion in 995 healthy subjects with normal levels of serum lipase and amylase using secretin‐enhanced magnetic resonance cholangiopancreatography (sMRCP). They found that the average secretion of the group aged 20‐29 years and the group aged 80‐89 years was 10.8 and 7.3 mL/min for males, and 10.2 and 6.7 mL/min for females, respectively. The mean secretion of the elderly group fell by about 30% compared with the younger group.^29^ These studies all suggested that pancreatic reserve exocrine function was also significantly reduced in the elderly.

## 5 CONCLUSION

Recent studies have proved that aging is accompanied by a degeneration, to a variable degree, in pancreatic structure (i.e. volume, weight, shape, microstructure and ultrastructure), as well as in both endocrine and exocrine function, and that this is one of the reasons for the growing morbidity of pancreatic diseases and conditions such as dyspepsia and diabetes mellitus. This finding also provides a theoretical basis for the clinical application of pancreatin in the treatment of dyspepsia in the elderly.^30^ It needs to be kept in mind that, although the aging pancreas still possesses an effective compensatory capacity that prevents the elderly from suffering from pancreatic insufficiency to some degree, it is a fragile balance, one that is influenced by the internal and external metabolic environment of the body, which may tend to result in pancreatic diseases as the body ages. Further studies on the mechanism of pancreatic aging and targeted interventions to prevent the elderly from suffering pancreatic diseases or pancreas‐related diseases will be needed in the future. Age‐related changes in pancreas are illustrated in Table 1.

**Table 1:** Age related changes in pancreas

Aspects		Age related changes of the pancreas
Morphological changes	Macrostructure	Decreased quality of aging pancreas^1^; Shrinking volume decreasing from 78.85 cm^3^ at the age of 70‐80 y^2^; Increased hardness^3^; Dilatation of the pancreatic duct, the mean diameter of which increased from 1.5 mm to 2.3 mm.^4^
	Microstructure	Increased fibrosis around the acini, islets and extracellular matrix^9^; Disrupted islets architecture and scattered islets can be found^10^; Apparent fatty infiltration, papillary projections into the lumen of the ducts.^8^
	Ultrastructure	Mitochondrial dehydration and swelling^13^; Evident vacuolization^13^; Increased lipid droplets and lysosomes^13^; Reduced enzymogen particles in pancreatic acinar cells and β cells^13^; Autophagosomes are observed.^15^
Exocrine function	Deteriorated basic exocrine function proved by dynamic magnetic resonance imaging of pancreatic biliary flow^26^; Decreased reserved exocrine function: duodenal fluid production after intravenous injection of secretin increased until 43 y of age, and then began to decline^28^; Increased incidence of EPI and SEPI, which were 13.4% and 5.6% in the group aged 70‐75 y, while 6.0% and 3.0% in the group aged 50‐54 y.^23^	
Endocrine function	Down‐regulated islet cell function with aging: reduced GSIR (glucose‐stimulated insulin release) and PSIR (palmitic acid‐stimulated insulin release) accompanied by the decrease of the insulin secretion peak and a gradually delayed peaking time, manifesting as obvious impaired glucose tolerance.^16^, ^17^	

## CONFLICTS OF INTEREST

Nothing to disclose.

## AUTHOR CONTRIBUTIONS

*Writing of paper*: both authors. *Design, literature review, coordination*: Songbai Zheng. *Literature reading, linguistic revision* *, design and drawing of the table*: Lu Wang.
